# Supplementary material for: Modelling and assessing additional transmission routes for porcine reproductive and respiratory syndrome virus: Vehicle movements and feed ingredients
Source: Transbound Emerg Dis. 2022 Mar 3;69(5):e1549–60. doi: 10.1111/tbed.14488 (PMC9790477; doi:10.1111/tbed.14488)
Supplement: Supplementary file 1 — SUPPORTING INFORMATION [file TBED-69-e1549-s001.docx]

**Supplementary Material**

**Modelling and assessing additional transmission routes for porcine reproductive and respiratory syndrome virus: vehicle movements and feed ingredients**

Jason A. Galvis ^1^, Cesar A. Corzo^2^, Gustavo Machado^1^*

^1^1 Department of Population Health and Pathobiology, College of Veterinary Medicine, North Carolina State University, Raleigh, NC, USA.

^2^Veterinary Population Medicine Department, College of Veterinary Medicine, University of Minnesota, St Paul, MN, USA.

***Corresponding author:** [gmachad@ncsu.edu](mailto:gmachad@ncsu.edu)

***Section 1. A descriptive analysis of model parameters used in the PRRSV simulated transmission.***


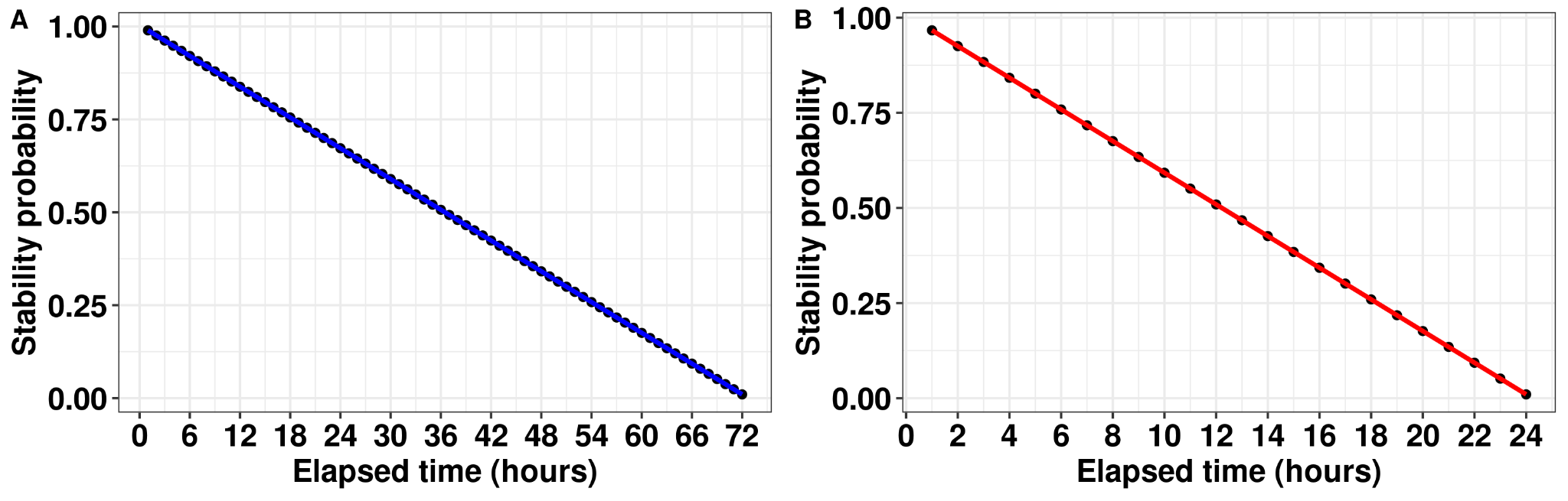


**Figure S1**. The distribution of PRRSV stability probability on vehicle surface over time. In A), we show the cold season decay which included the months between October to March, and B) warm season decay in PRRSV suitability which included the months between April to September. In summary, here we assumed that PRRSV suitability decreased linearly over time after a vehicle visited a farm, down to zero after 72 hours for cold months and 24 hours for warm months, when PRRSV was no longer viable. This approach was used for all vehicle related contact networks including vehicles transporting feed, pigs-to-farm, pigs-to-market and crew.


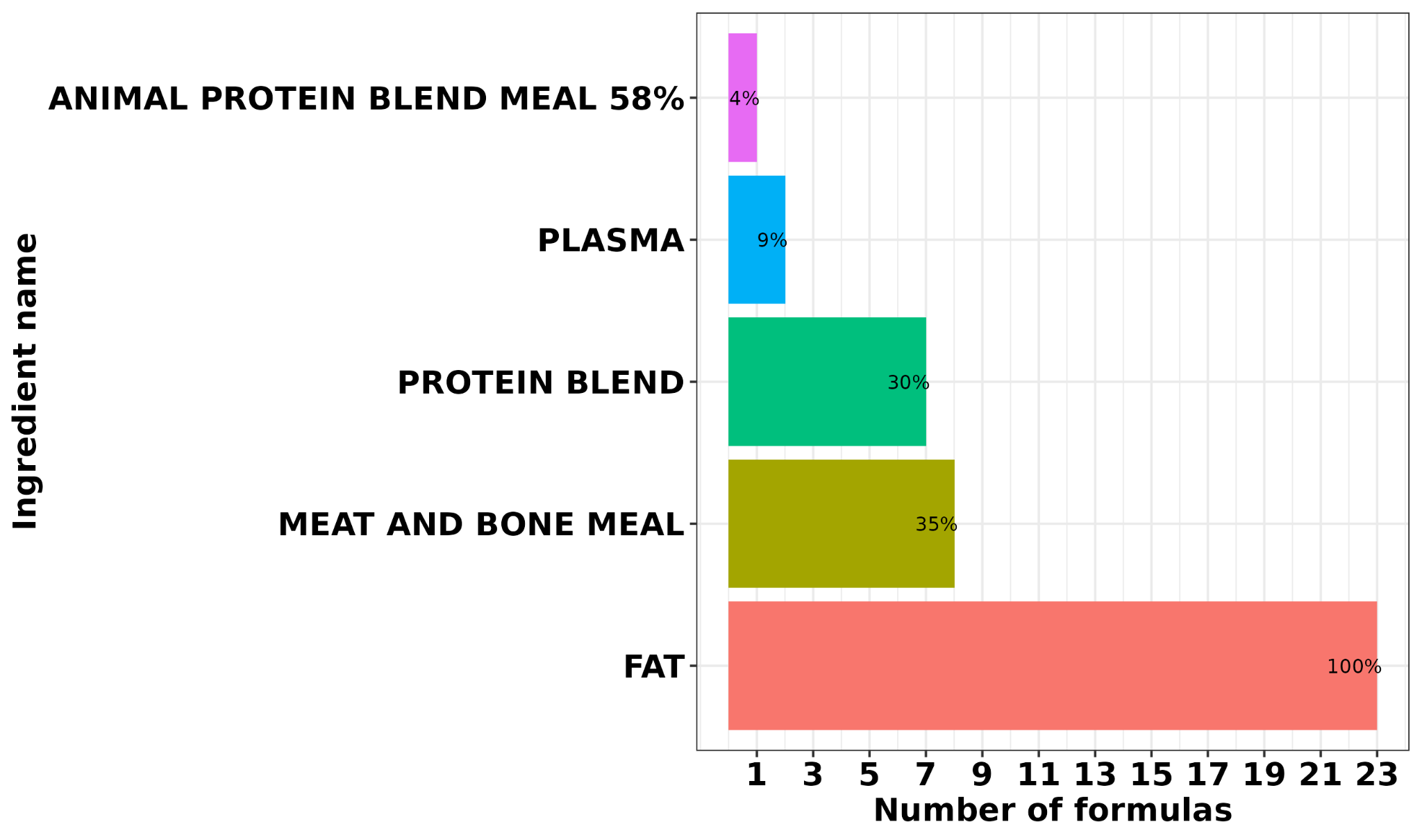


**Figure S2.** The overall proportion of animal by-products delivered to farms from January 2020 until December 2020. The x-axis shows the number of unique feed formulations and the y-axis each animal by-product utilized by company A.


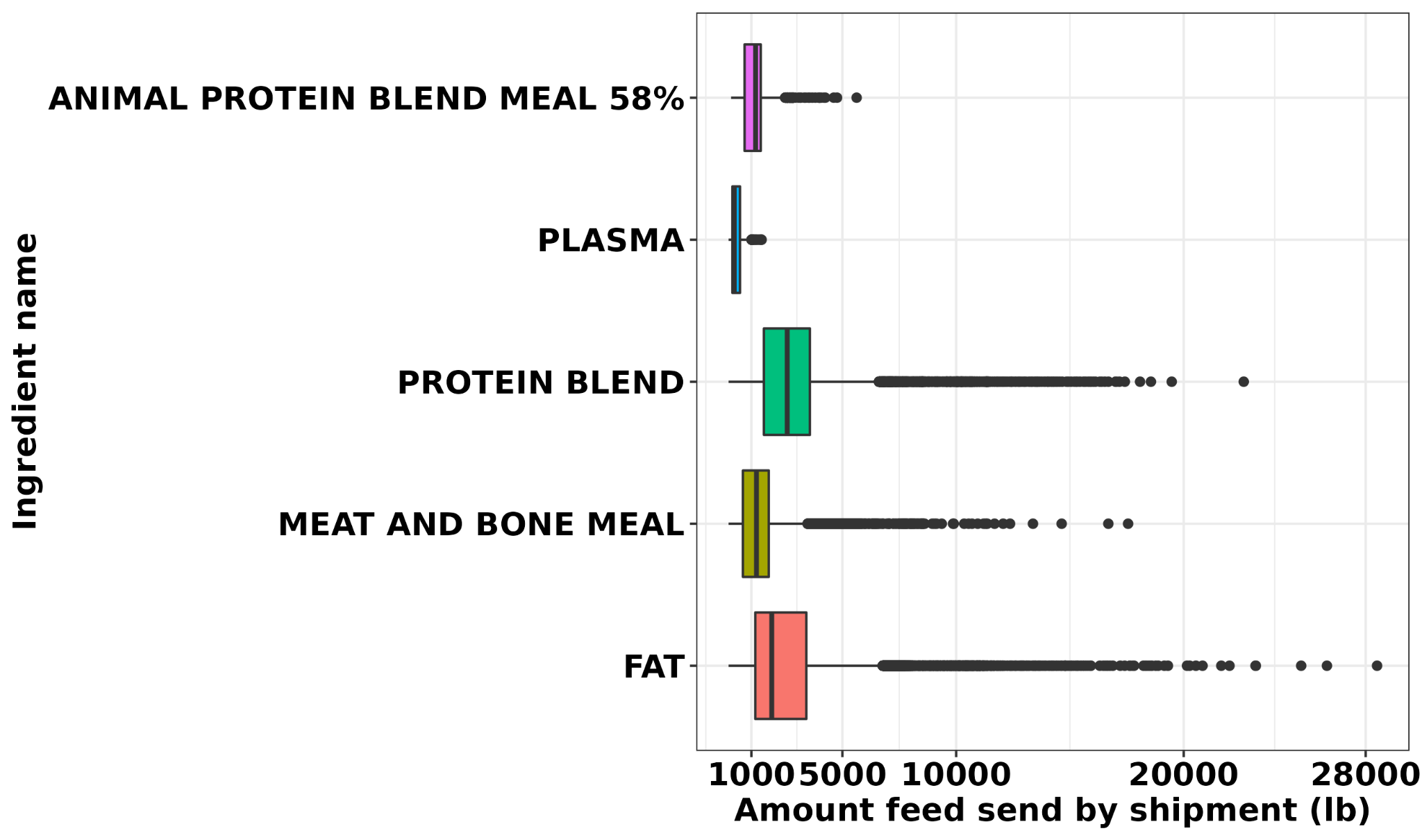


**Figure S3.** The weight (lb) distribution of animal by-products in shipments of feed formulation delivered to the farms of company A in the study area over from January 2020 until December 2020.

**Table S1.** Terminology and definition from the social network analysis.

| **Network terminology** | **Definition** | **Reference** |
| --- | --- | --- |
| Node | Element of the network representing the farms. | - |
| Edge | Link among two nodes. | - |
| Static network | Once an edge exists between two nodes, it is present for the whole time period. | (Kao et al., 2007) |
| Temporal network | The edges between two nodes only exist at different time steps. | (Lentz et al., 2016) |
| Density | Represent the proportion of edges among nodes in the network that are actually present. | (Wasserman and Faust, 1994) |
| Causal fidelity | Quantify the error of the static representation of a temporal network by comparing the number of paths in the static and temporal networks. | (Lentz et al., 2016) |
| Strongly Connected Component (SCC) | It is a subset of nodes for which a *directed* path exists between all pairs of them allowing them to be mutually accessible by following the direction of the links in the network. | (Lentz et al., 2016) |
| Larger Strongly Connected Component (LSCC) | It is the larger number of nodes in a strong connected component. | (Lentz et al., 2016) |
| In-degree | Number of nodes providing animals to a specific node. | (Wasserman and Faust, 1994) |
| Out-degree | Number of nodes obtaining animals from a specific node. | (Wasserman and Faust, 1994) |
| Betweenness | The frequency at which a node is in the shortest path between pairs of other nodes in the network. | (Freeman, 1978) |
| Ingoing contact chain (ICC) | Subsets of nodes that can reach a specific node by direct contact or indirect contacts through a sequential order of edges through other nodes using the temporal network. | (Nöremark and Widgren, 2014) |
| Outgoing contact chain (OCC) | Subsets of nodes that can be reached by a specific node by direct contact or indirect contacts through a sequential order of edges through other nodes using the temporal network. | (Nöremark and Widgren, 2014) |

To calculate the barrier index (vegetation level, utilized to modulate the probability of local transmission), we used a linear regression, to express PRRSV infected farms from 2020 as a function of the Enhanced Vegetation Index (EVI) and yearly seasonality (spring, summer, fall and winter). We found that PRRSV frequency decreased as EVI increased, with a stronger association in winter and fall seasons (Figure S4). Here we used the regression coefficients to predict weekly PRRSV incidence, which then were transformed into parameter *a*, which was scaled into values between [0, 1], later utilized to modulate the local transmission.


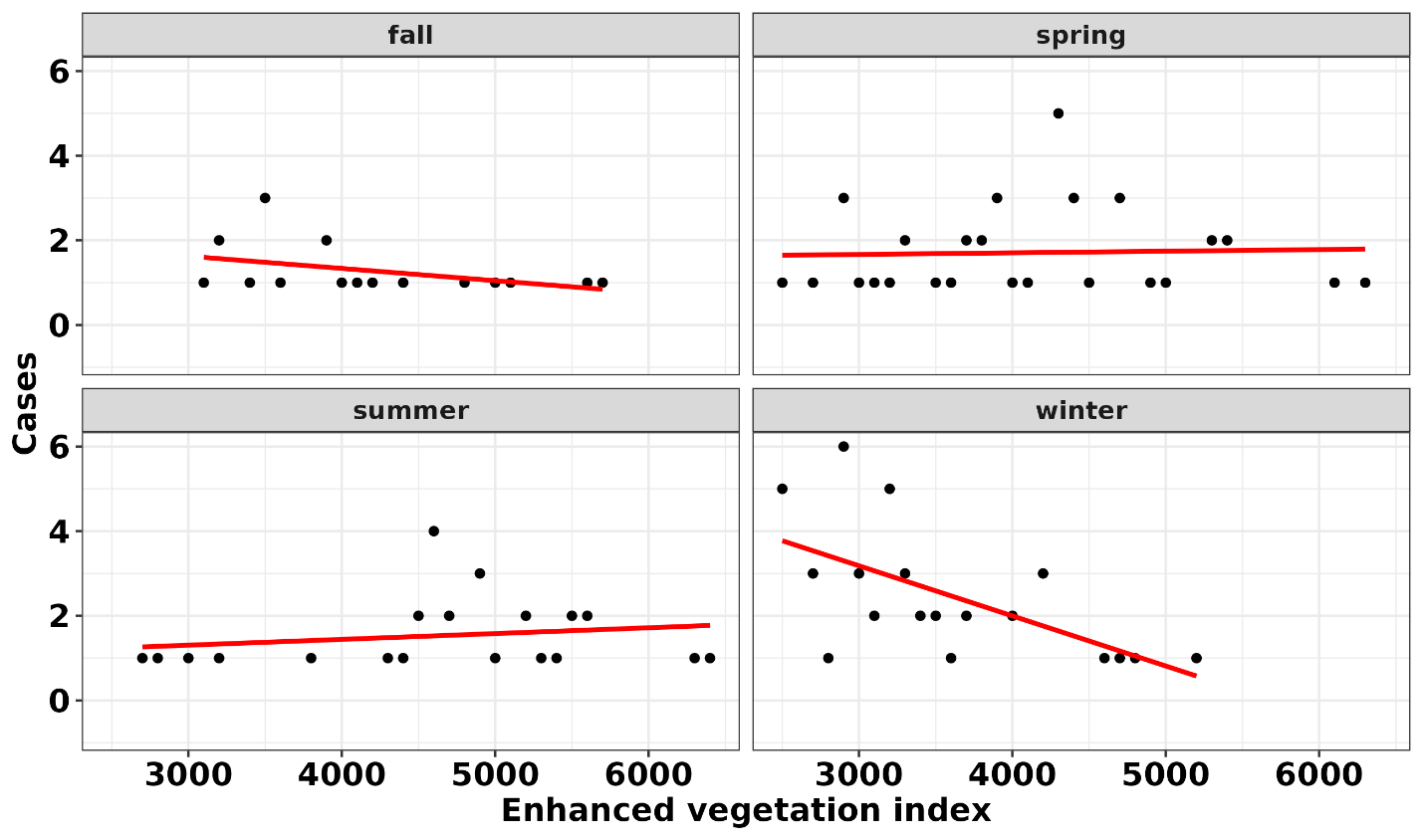


**Figure S4**. Linear regression of PRRSV infected farms. The y-axis is the number of PRRSV outbreaks and in the x-axis EVI. Fall: coefficient -0.00029, standard error 0.00018, *p*-value .12, Spring: coefficient -0.00001, standard error 0.00023, *p*-value .96, Summer: coefficient 0.00013, standard error 0.00018, *p*-value .46. Winter: coefficient -0.00103, standard error 0.0004, *p*-value .02.


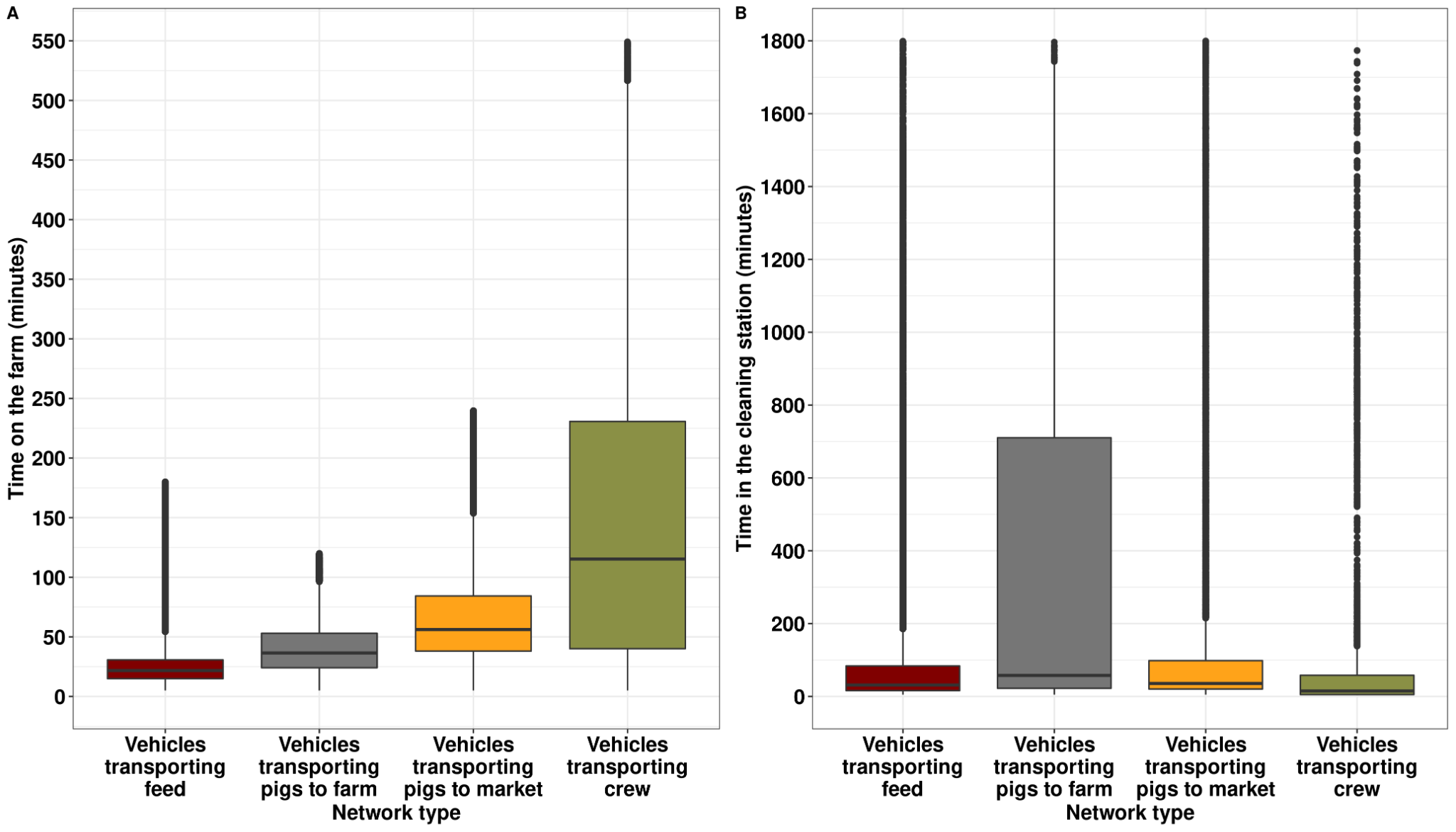


**Figure S5**. The time vehicles spent on each farm visit. The boxplot shows the distribution in minutes that each vehicle remained within farms premises in A) and at cleaning stations in B).


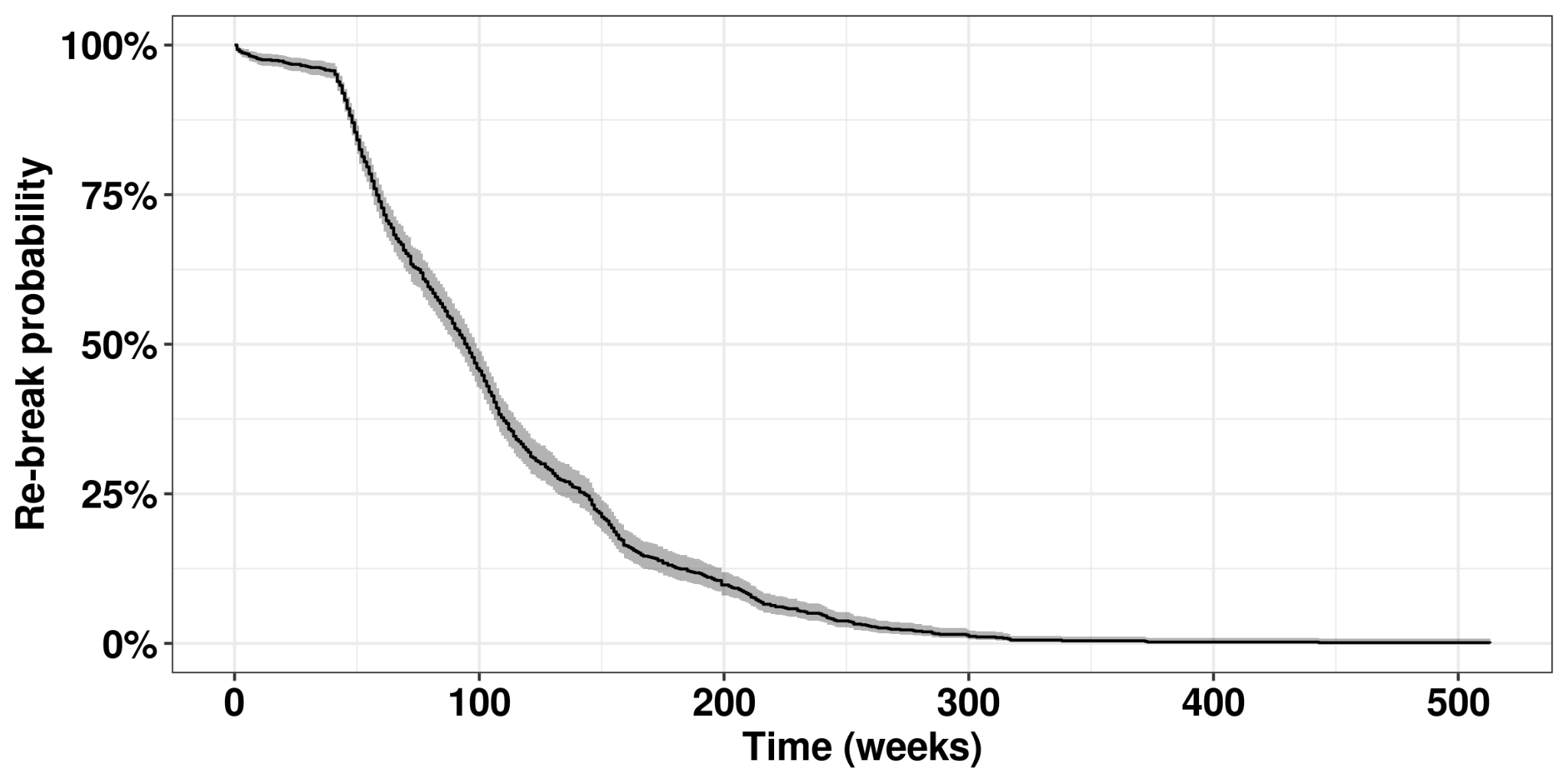


**Figure S6**. A survival analysis of infected and recovered farms from 2009 to 2019. In this example we show the distribution used for each farm to calibrate the re-break probability


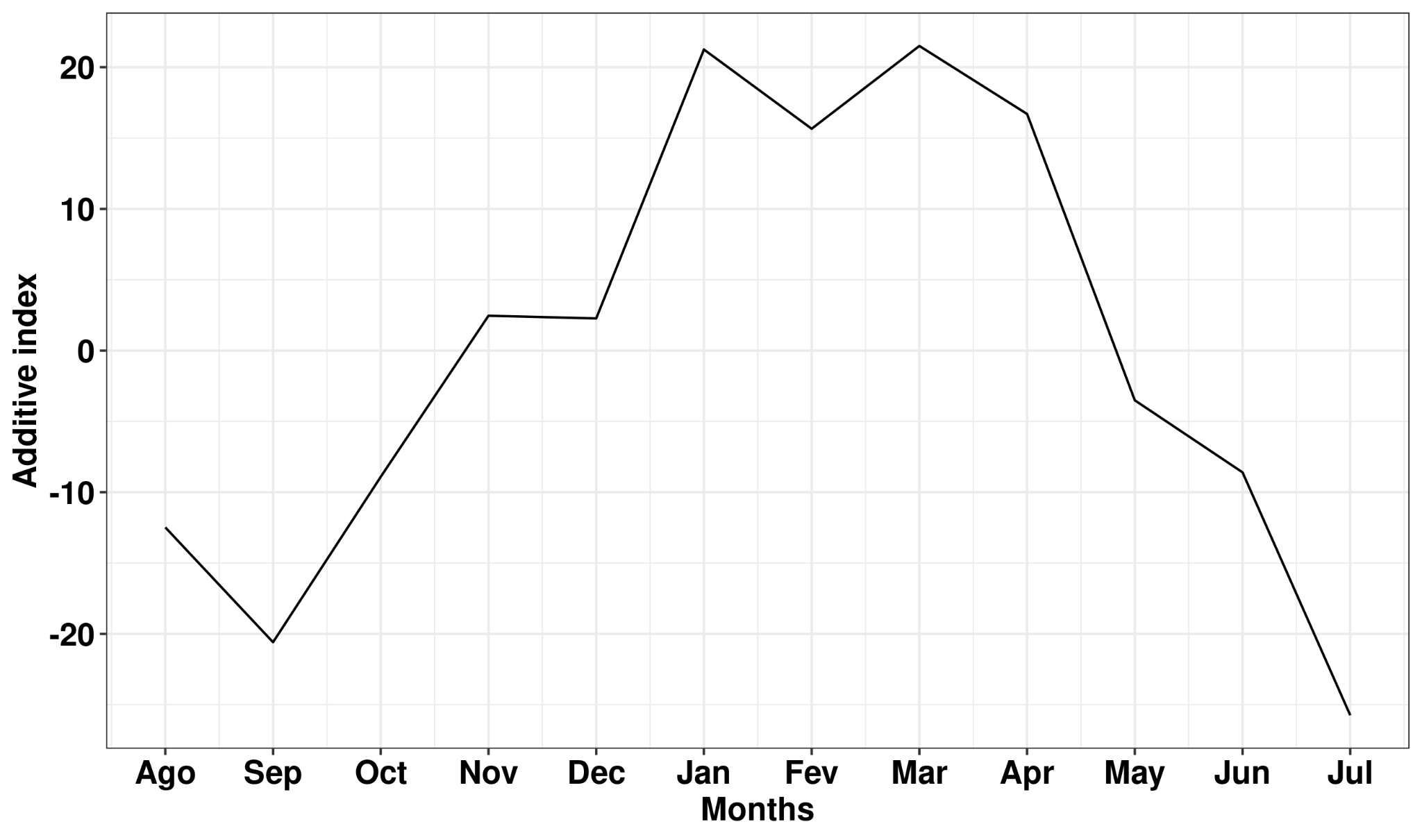


**Figure S7**. Monthly seasonality index calculated from the frequency of PRRSV, calculated by an additive moving average decomposition derived from analysis of the PRRSV records from 2015 to 2019.

***Section 2: Model calibration and main model outputs***

The model is calibrated in two steps, first fitting the frequency of observed outbreaks on time, and second fitting the location of the observed outbreaks. In Table S2 we show the summary statistics used in step 1 of the Approximate Bayesian Computation (ABC) rejection algorithm, where the tolerance interval represents the square error allowed from the simulation to the observed values. In the model calibration, a particle is defined as a set of transmission parameters (Table S3). For the model calibration in step 1, we defined as an accepted particle the set of transmission parameters, whose simulation results are lower than the tolerance interval for all the variables when compared with the observed values. Thus, the range of error accepted (tolerance interval) defines the number of particles accepted in the step 1 of the model calibration. In addition, low tolerance interval values produce particles with similar results to the observed values, but at the same time, the number of particles accepted decreases. Thus, in this study the tolerance interval values were chosen through initial exploratory analysis, selecting the minimum tolerance interval values to accept an adequate number of particles to be analyzed in the step 2 of the model calibration, in this case for each 100,000 particles tested around 20 were accepted in the step 1.

**Table S2.** Summary statistics used by the ABC Sequential Monte Carlo rejection algorithm for the model.

| **Summary statistics** | **Observed values** | **Tolerance interval (ϵ)** |
| --- | --- | --- |
| Total number of sow farms with detected cases | 96 | 20 |
| The weekly average number of sow farms with detected cases | 1.8 | 0.5 |
| The weekly maximum number of sow farms with detected cases | 9 | 5 |
| Total number of nursery farms with detected cases | 37 | 20 |
| The weekly average number of nursery farms with detected cases | 0.7 | 0.5 |
| The weekly maximum number of nursery farms with detected cases | 6 | 5 |
| Total number of finisher farms with detected cases | 17 | 20 |
| The weekly average number of finisher farms with detected cases | 0.3 | 0.5 |
| The weekly maximum number of finisher farms with detected cases | 2 | 5 |
| Expected prevalence in finisher and nursery farms | 30% (expert opinion) | 100 |

To assess the model performance, we evaluated the probability to predict cells (10 x 10 km squares) with true infected cells (cells where at least one sow farm outbreak was recorded) at time *t*. Each sow farm was allocated to a cell in the spatial grid (total of 154 cells in the study area). For each particle accepted in the step 1 model fitting, the risk of each cell was calculated by the sum of times at least one farm within a cell was identified with infected status after 100 simulations; we utilized a percentiles thresholds (r) approach to determine cells at high and low risk, where high risk cells were compared with the true infected cells at time *t*. Subsequently we estimate the model sensitivity and specificity, for all thresholds, as follows:

S_r_ = TP_r_/(TP_r_ + FN_r_)

E_r_ = TN_r_/(TN_r_+FP_r_)

where true positives (TP) was the subset of cells with observed outbreaks and the estimated risk was above the r threshold; false negatives (FN) was the subset of cells with observed outbreaks and the estimated risk was below the r threshold; true negative (TN) was the subset of cells without observed outbreaks and the estimated risk was below the r threshold; and false positives (FP) was the subset of cells without observed outbreaks and the estimated risk above the r threshold.

In step 2 of the ABC rejection algorithm, the sensitivity and specificity were calculated for each particle accepted in the step 1 of model fitting. The particles accepted were those with sensitivity values ≥30% with r = 85th and ≥50% with r = 70^th^ (percentile and sensitivity thresholds values were chosen arbitrarily after authors discussed the minimum performance of the model). The priors for each parameter were drawn from a uniform distribution that ranged between 0 and 1.5 for pig movements transmission rate, 0 and 0.001 for local transmission, the four transporting vehicles and amount of animal fat and meat and bone meal in the feed meals transmission rates, 0 and 0.01 for re-break transmission rate, 0 and 1 for farm’s biosecurity in sow farms and finally between 0 and 0.09 for maximum effective surveillance in nursery, finisher and other farms. These range values were chosen according to model performance to fit the temporal and spatial distribution of PRRSV cases through some test simulations, thus reducing the number of simulations and processing time in the model calibration. It is worth noting that farm’ biosecurity was only included for sow farms, thus there are no parameters representing nursery, finisher or other farms. Finally, here we were not able to describe the number simulation necessary to accept particles, similar how it was described in step 1, because we did not track the number of simulation used in step 2.

**Table S3.** List of transmission parameters used in the simulations and values estimated from the posterior distribution of the 100 particles accepted in the model calibration.

| **Model parameter** | **Symbol** | **Average values** | **95% credible interval*** | **Details & references** |
| --- | --- | --- | --- | --- |
| Transmission rate of pig movements | β_n_ | 0.428 | 0.11-1.29 | ABC fitting |
| Local transmission rate | β_l_ | 0.00055 | 0.0001-0.0009 | ABC fitting |
| Transmission rate of vehicles transporting feed | β_f_ | 0.000014 | 0.0000004-0.00004 | ABC fitting |
| Transmission rate of vehicles transporting pigs to farms | β_p_ | 0.00026 | 0.000006-0.0009 | ABC fitting |
| Transmission rate of vehicles transporting pigs to market | β_m_ | 0.00049 | 0.00003-0.0009 | ABC fitting |
| Transmission rate of vehicles transporting crew | β_c_ | 0.00027 | 0.00003-0.0008 | ABC fitting |
| Transmission rate of animal fat in the feed meal | β_a_ | 0.00042 | 0.000002-0.0009 | ABC fitting |
| Transmission rate of meat and bone meal in the feed meal | β_b_ | 0.00042 | 0.000005-0.0009 | ABC fitting |
| Transmission rate of re-break | β_r_ | 0.0045 | 0.0001-0.009 | ABC fitting |
| Farm’ biosecurity | H(sow) | 0.57 | 0.02-0.99 | ABC fitting |
| Maximum effective surveillance | L(sow) | 0.95 | - | Expert opinion |
|  | L(nurseries) | 0.048 | 0.016-0.085 | ABC fitting |
|  | L(finisher) | 0.0015 | 0.0001-0.003 | ABC fitting |
|  | L(others) | 0.045 | 0.002-0.086 | ABC fitting |
| PRRSV seasonality | T | Weekly values calculated | - | Figure S7 |
| Average time for PRRSV detection | X0 | 4 weeks | - | Expert opinion |
| Average infectious time sow farms | - | 41 weeks | - | (Sanhueza et al., 2019) |

*Credible intervals calculated with method equal-tailed interval.

**
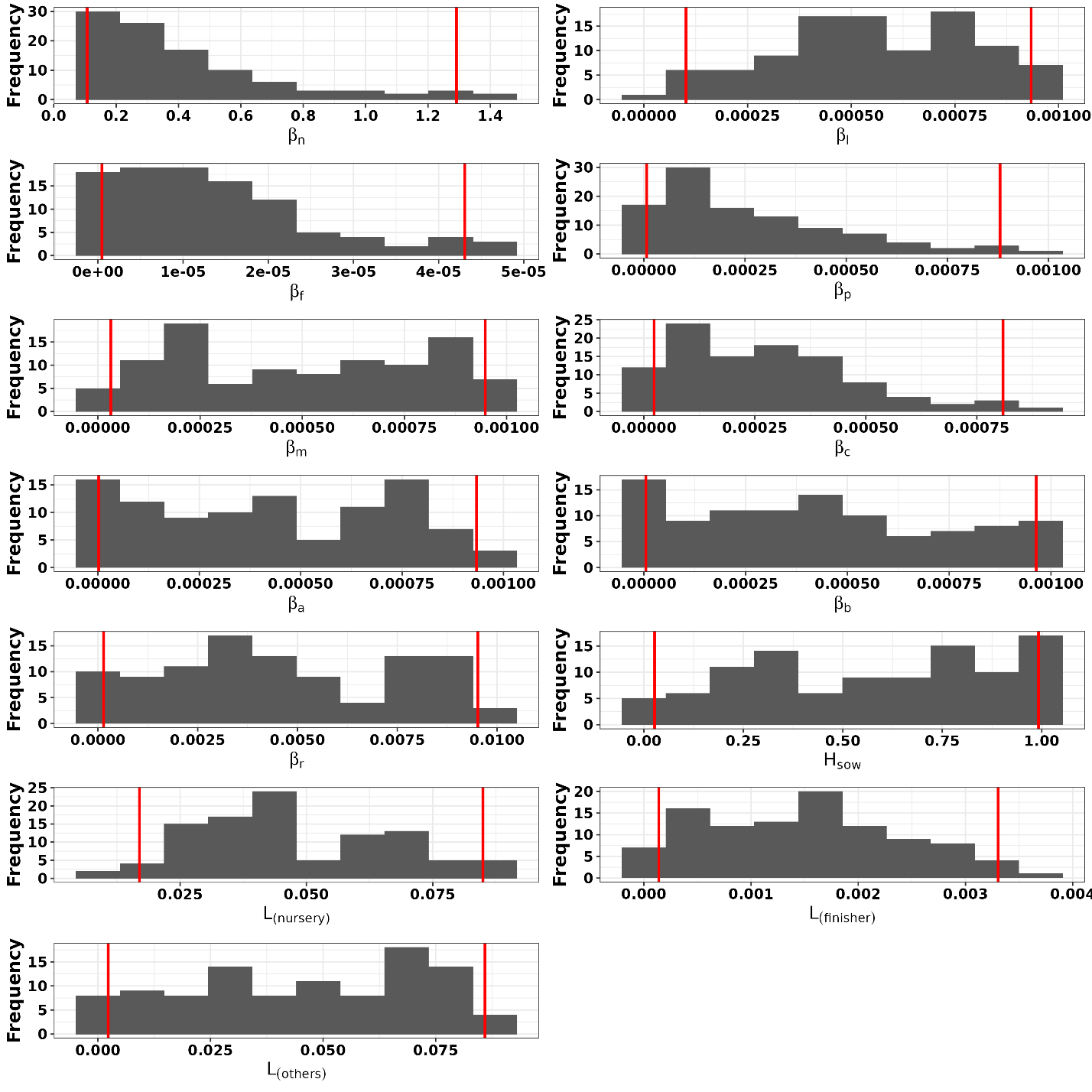
Figure S8.** Posterior distribution of the calibrated transmission parameters derived from 100 accepted particles, red lines represent 95% credible intervals calculated through equal-tailed interval method.


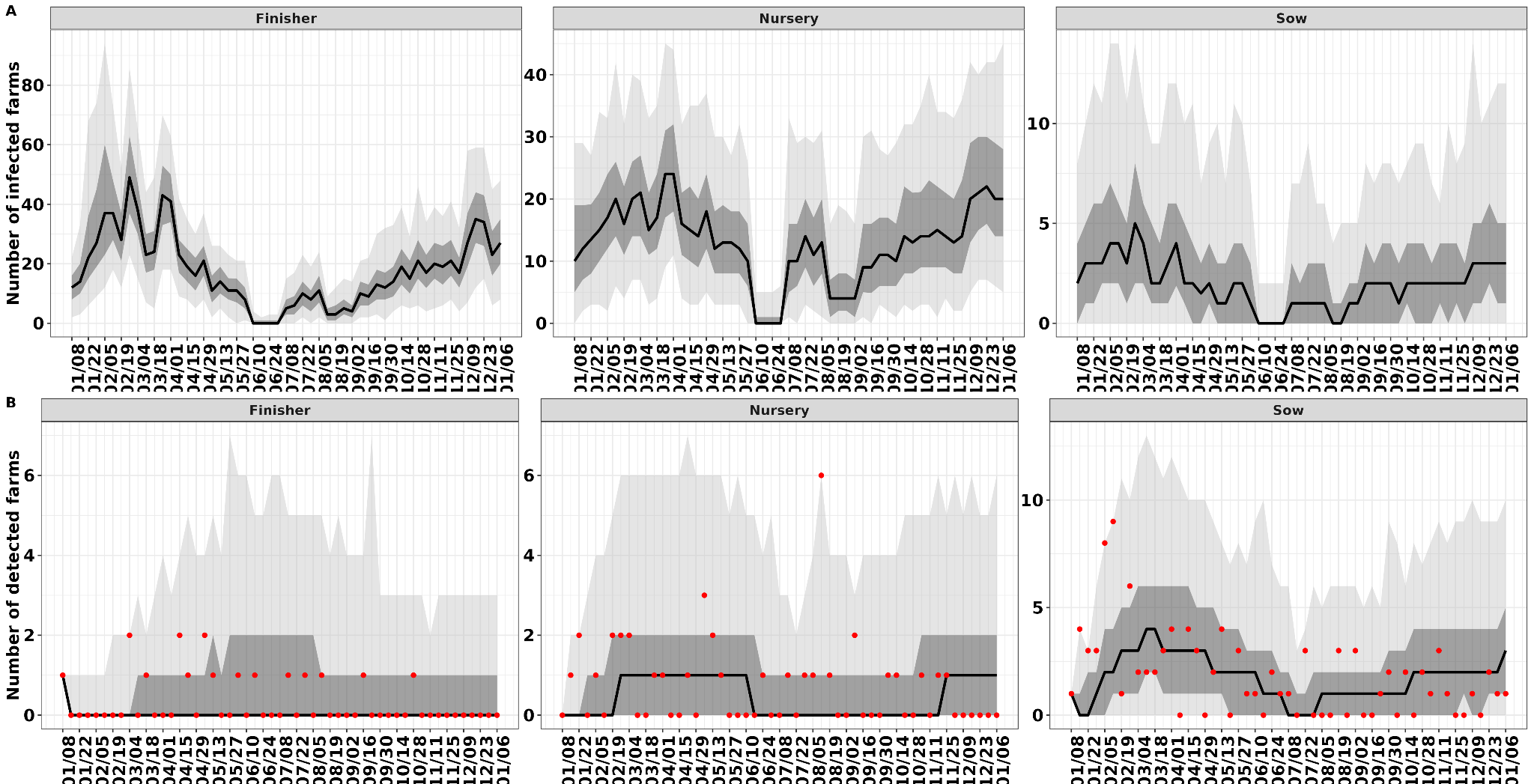


**Figure S9**. The simulated weekly number of infected farms in A) and infected detected farms (PRRSV outbreaks) in B). The black line represents the median, the dark shade areas represent a 75% credible interval and the light shade areas maximum and minimum generated by the model, and the red dots the frequency of true outbreaks reported in our data. Uncertainty in the estimated model parameters is reflected by 1,000 repeated simulations.


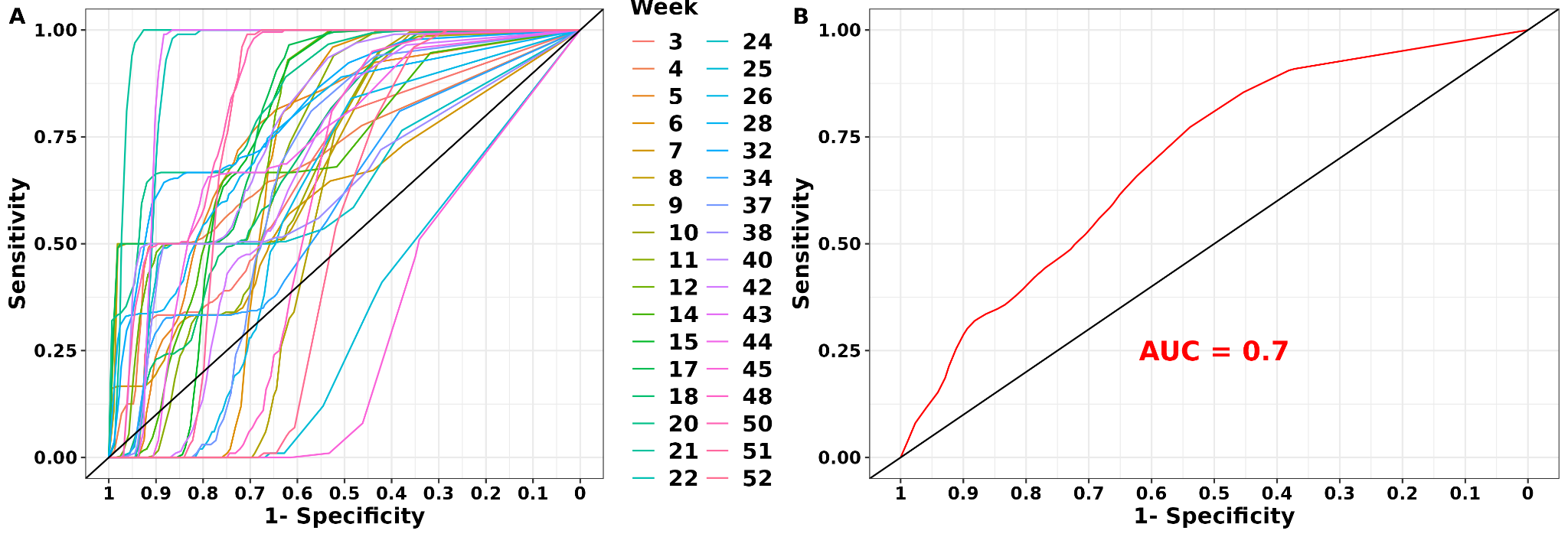


**Figure S10**. The average sensitivity and specificity for the weekly forecasts in A) and the average of all weeks in B). These values were calculated from 100 model calculations with each model calculation having 100 individual model iterations per predicted week to estimate the spatial location of observed outbreaks

***Section 3: Descriptive analysis of the between-farm pig movements and transportation vehicle movement networks, and the quantity of animal by-product in feed ingredients.***


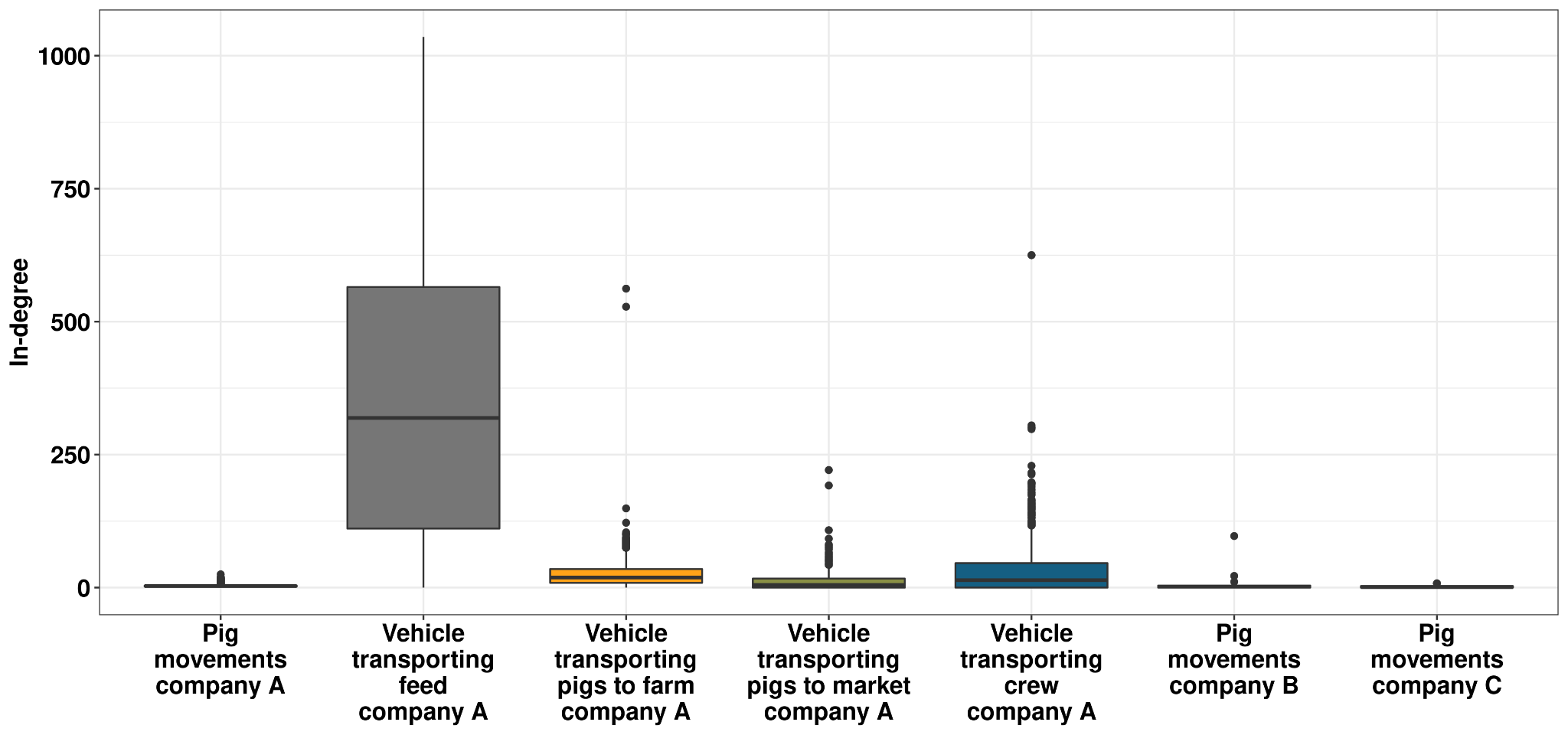


**Figure S11**. Boxplot with the distribution of in-degree for between-farm pig movements of each transportation vehicle movement networks.


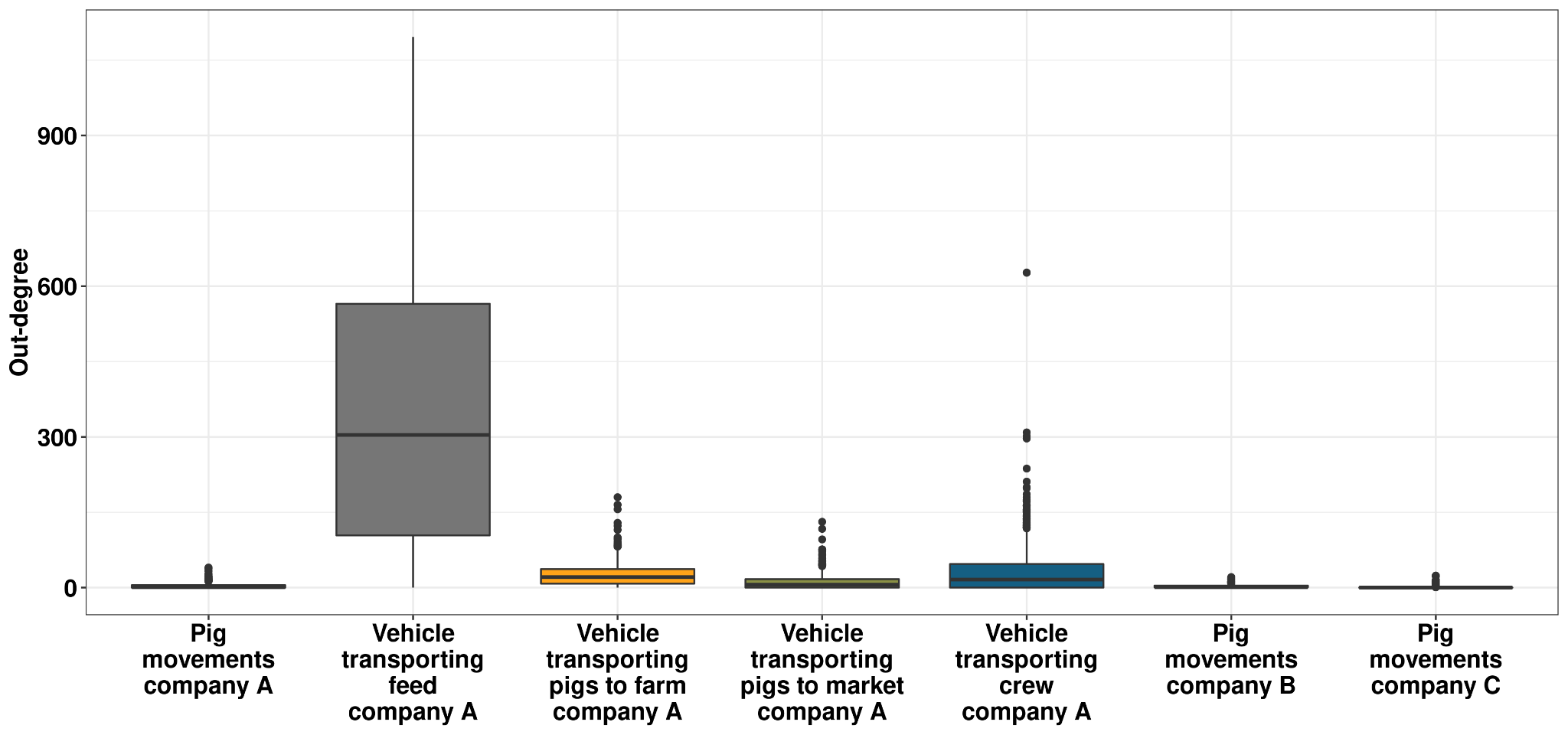


**Figure S12**. Boxplot with the distribution of out-degree for between-farm pig movements of each transportation vehicle movement networks.


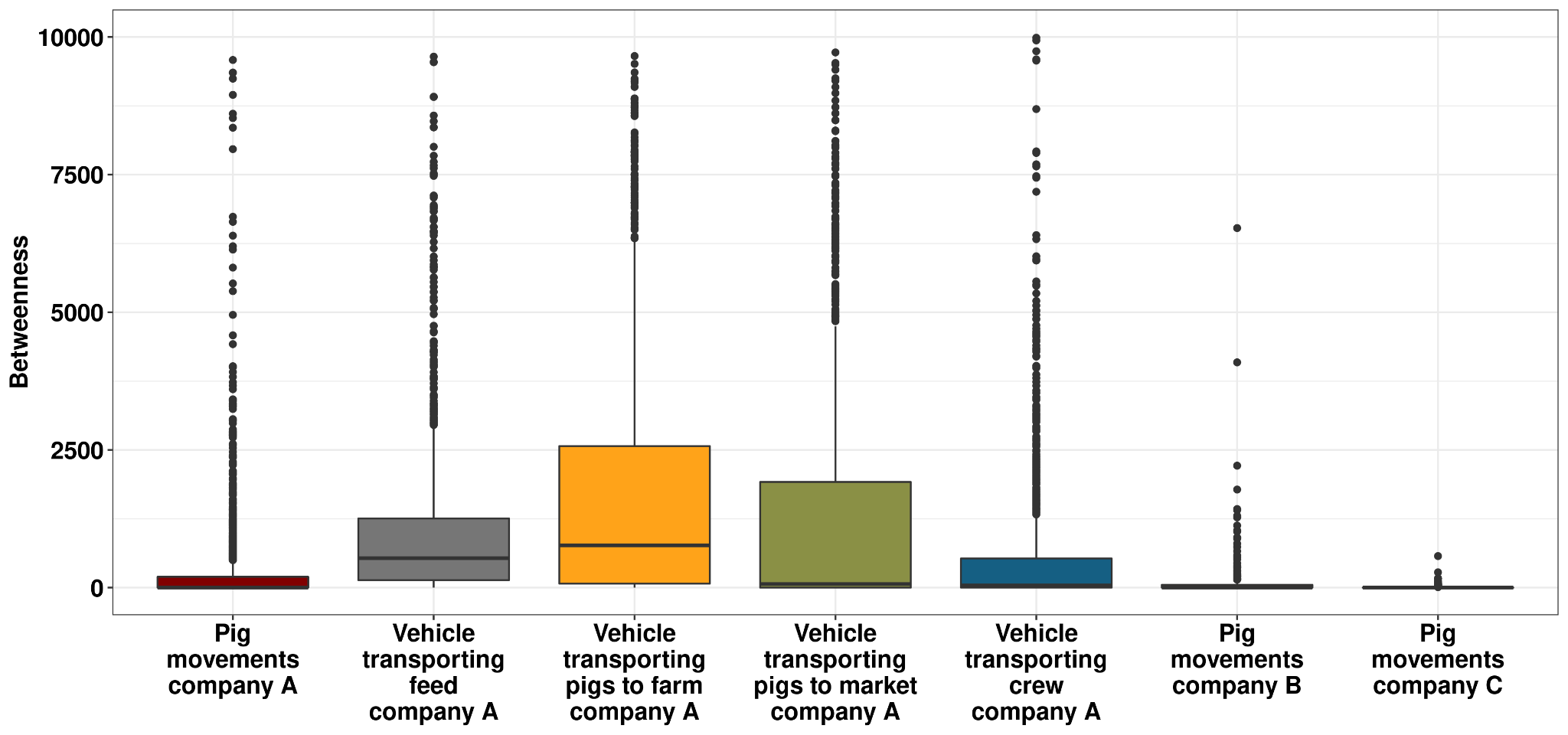


**Figure S13**. Boxplot with the distribution of betweenness for between-farm pig movements of each transportation vehicle movement networks.


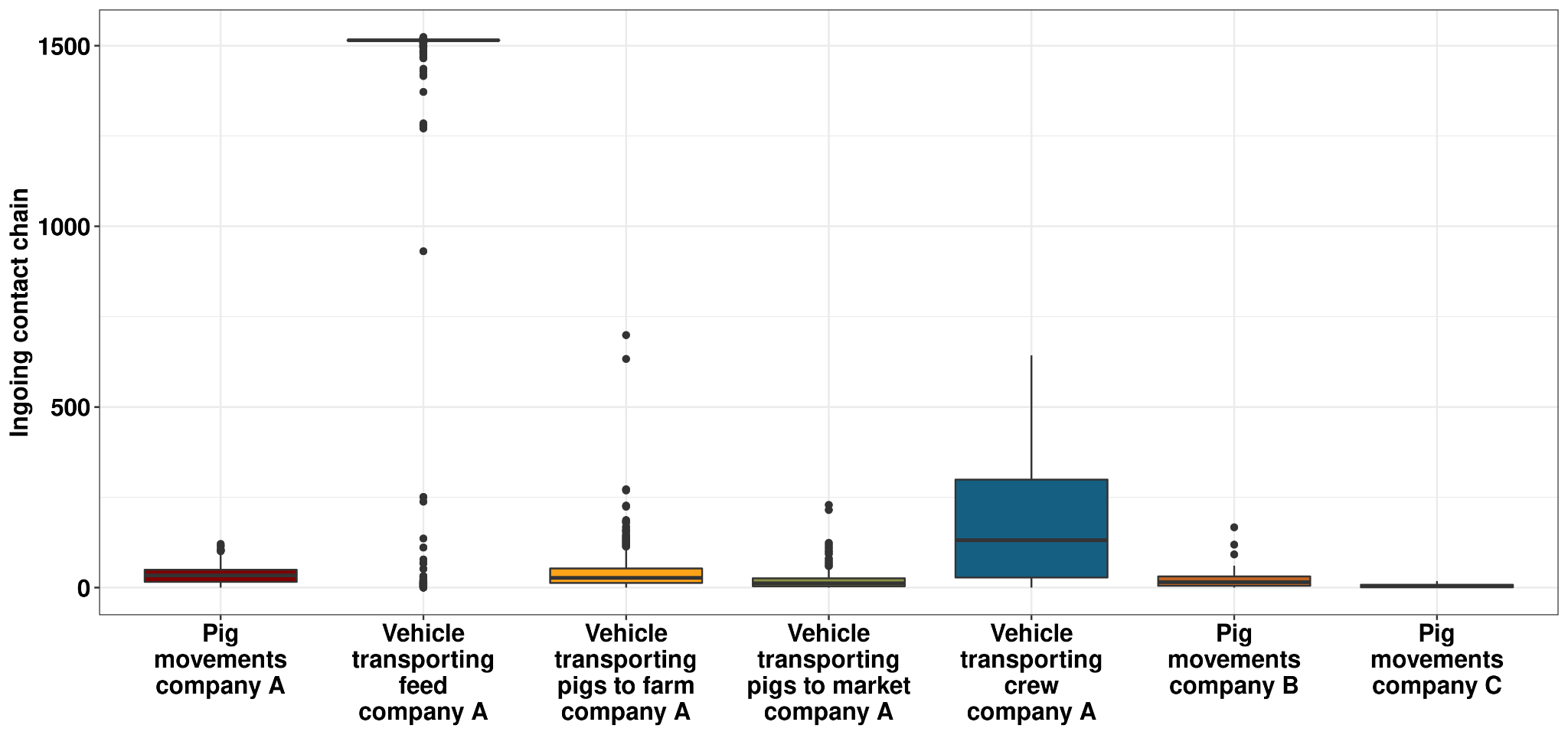


**Figure S14**. Boxplot with the distribution of ingoing contact chains for between-farm pig movements of each transportation vehicle movement networks.


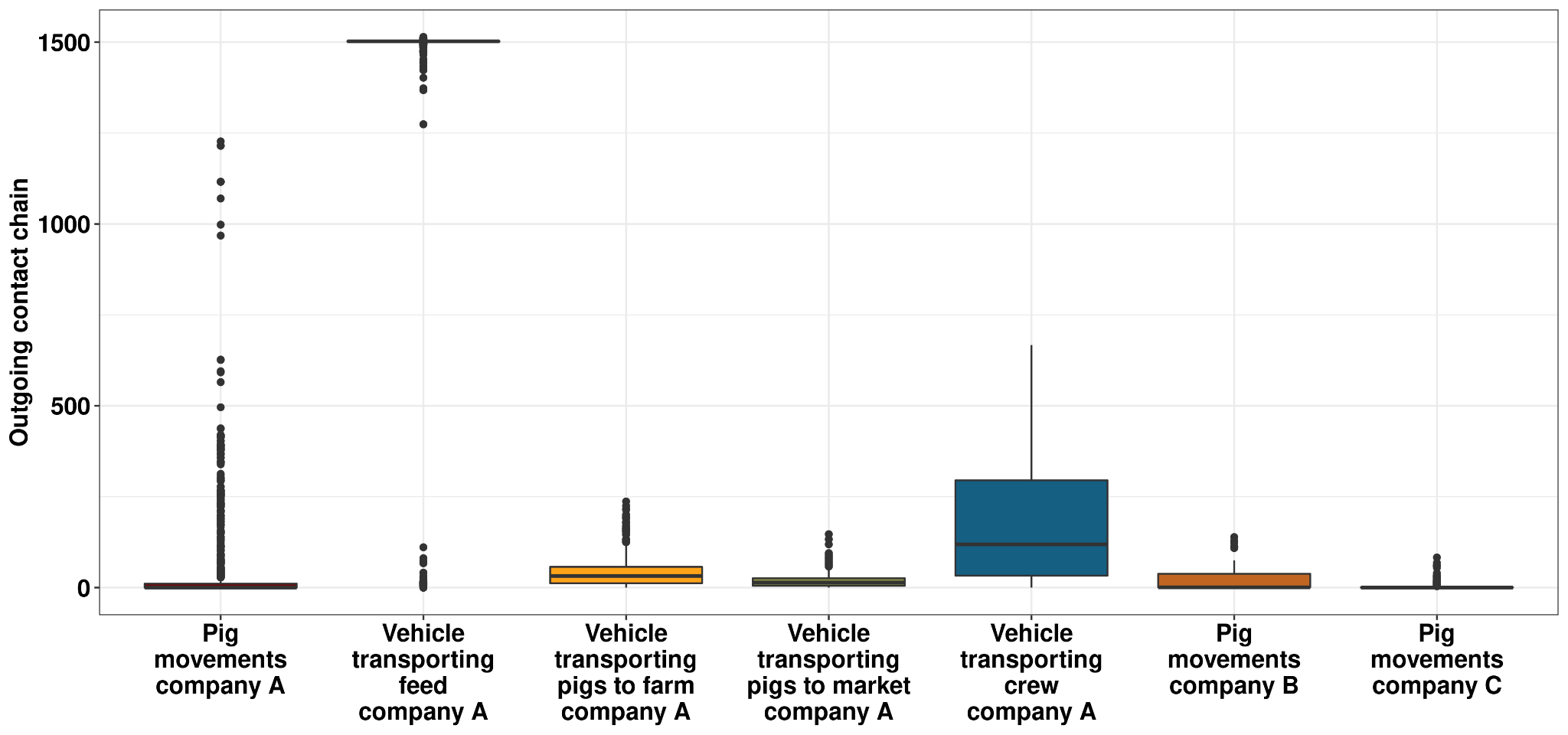


**Figure S15**. Boxplot with the distribution of outgoing contact chains for between-farm pig movements of each transportation vehicle movement networks.


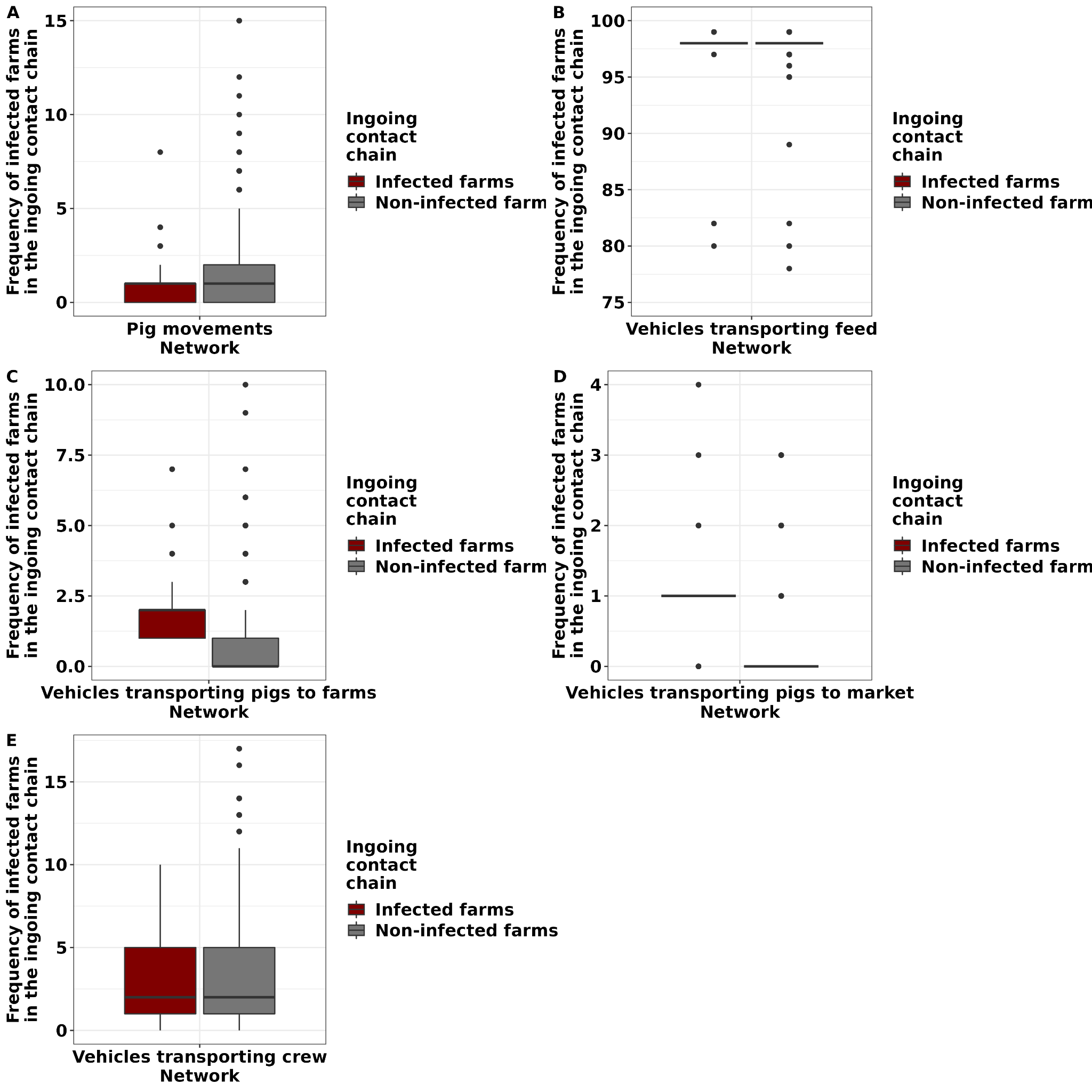


**Figure S16**. Boxplot comparing the frequency of infected farms in the ingoing contact chain of infected and non-infected farms of each transportation vehicle and pig movement networks. Infected farms are more frequent in the ingoing contact chain of other infected farms for the vehicles transporting feed, pigs to farms and pigs to market (Mann Whitney test *p* < .05). It is worth noticing that the statistical differences found among the groups can be influenced by the presence of outliers in the distribution, as well as the large number of farms analyzed (n = 2,294) that increase the test power and make it more likely to reject the null hypothesis.


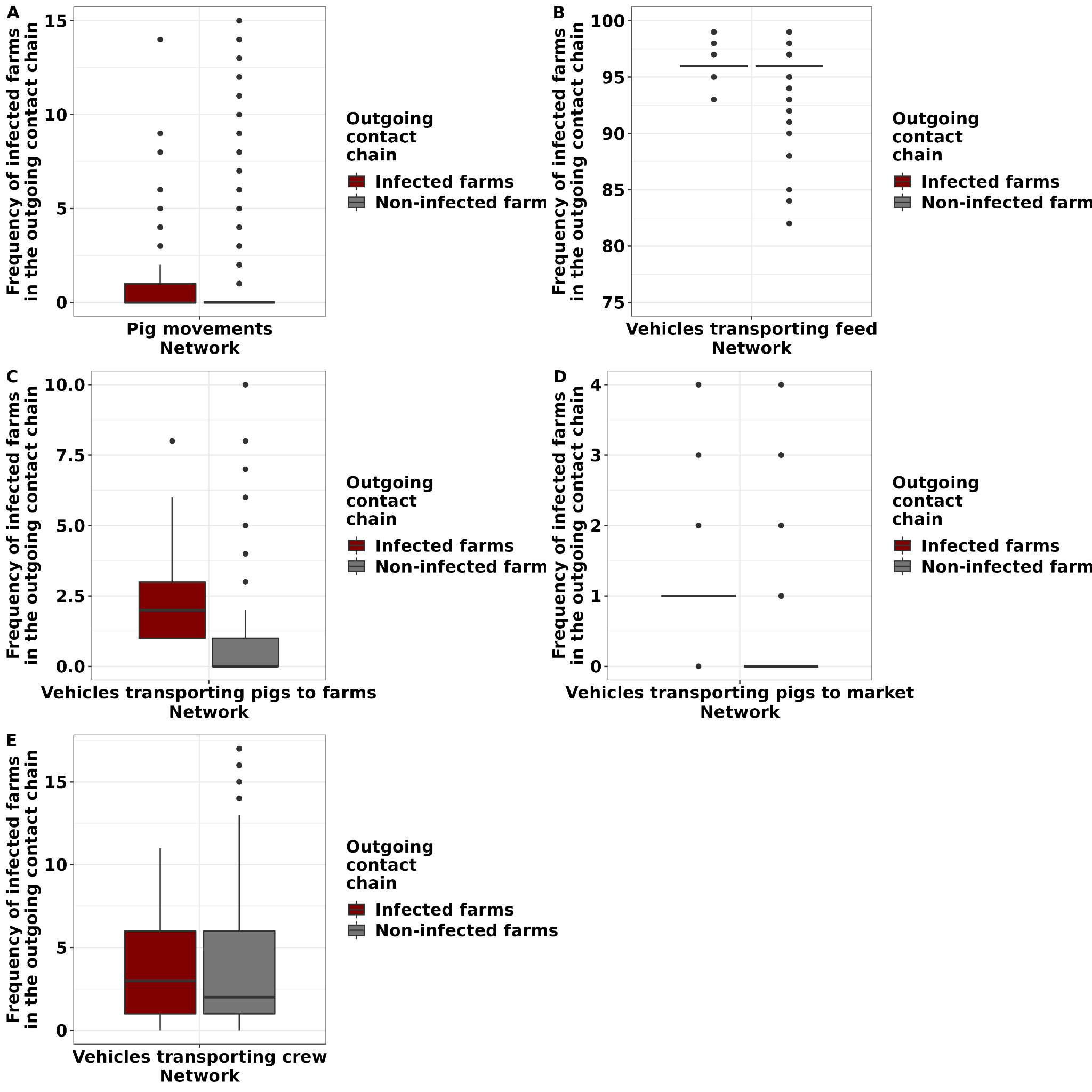


**Figure S17**. Boxplot comparing the frequency of infected farms in the outgoing contact chain of infected and non-infected farms of each transportation vehicle and pig movement networks. Infected farms are more frequent in the outgoing contact chain of other infected farms for pig movements and the vehicles transporting feed, pigs to farms and pigs to market (Mann Whitney test *p* < .05). It is worth noticing that the statistical differences found among the groups can be influenced by the presence of outliers in the distribution, as well as the large number of farms analyzed (n = 2,294) that increase the test power and make it more likely to reject the null hypothesis.


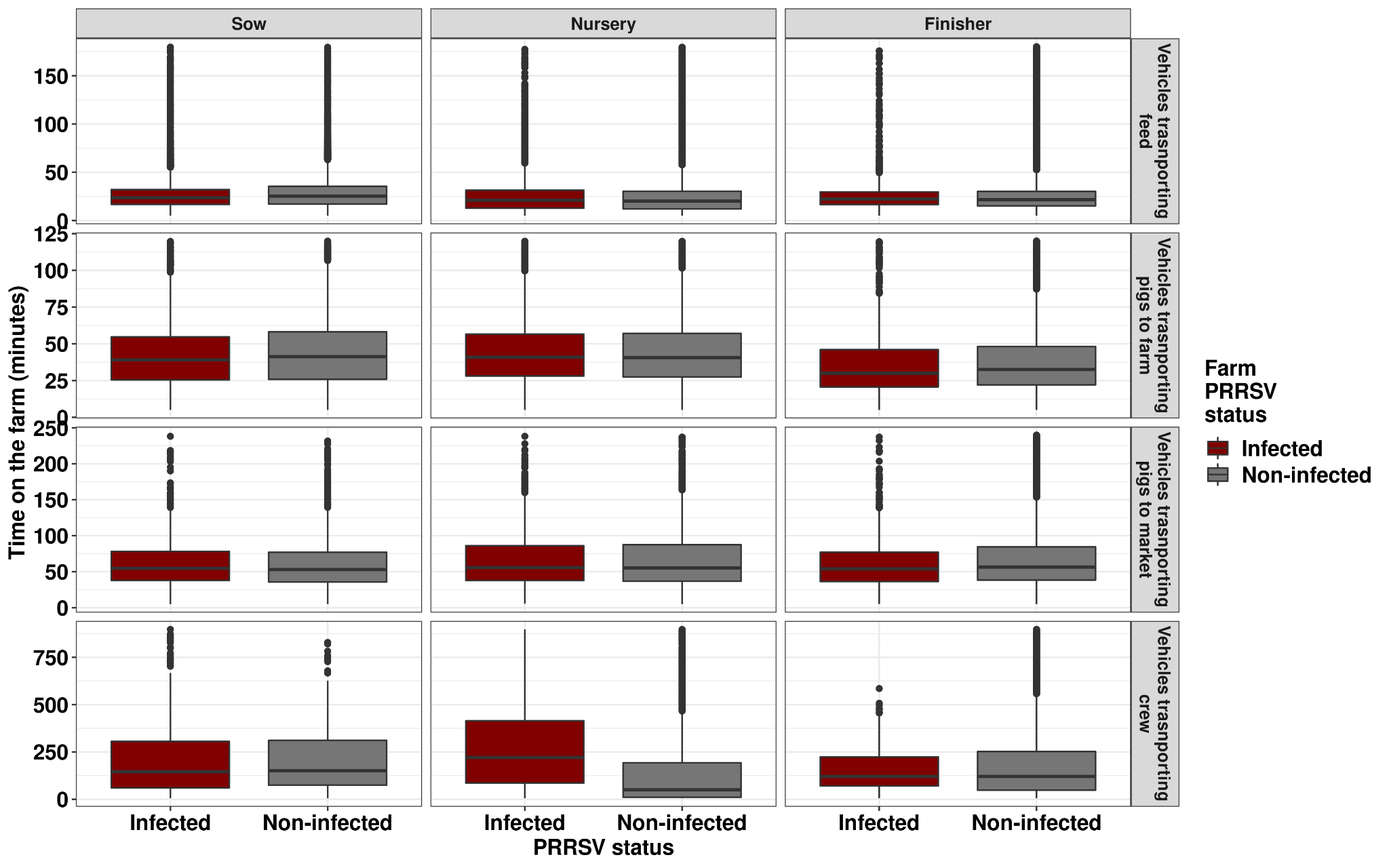


**Figure S18**. Boxplots compare the time vehicles remain on the farms of infected and non-infected farms for the different transportation vehicles (rows) and production types (columns). Vehicles transporting feed and crew to nursery farms were the only vehicles that showed a higher average of time on infected farms (Mann Whitney test *p* < .05).


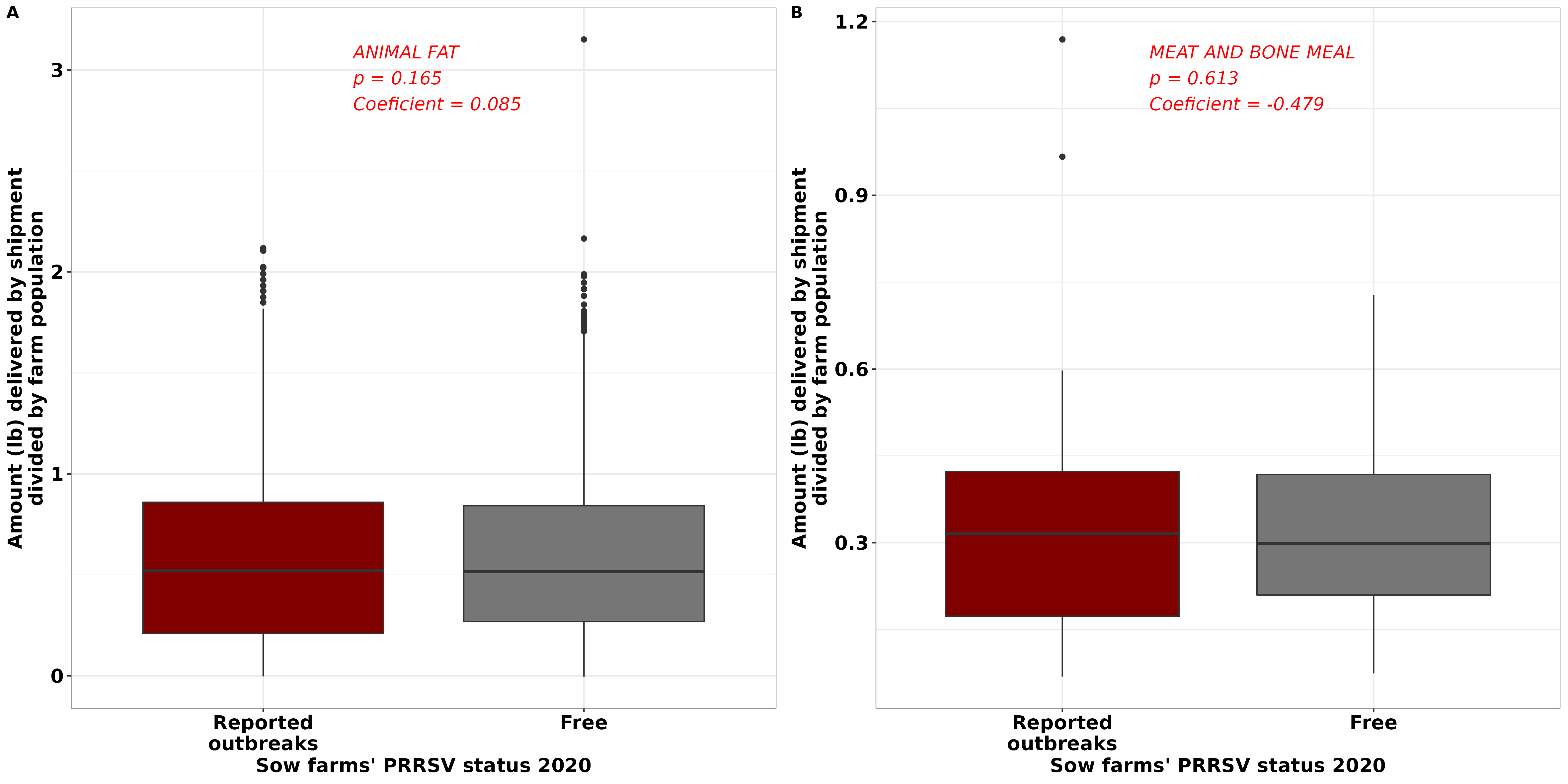


**Figure S19.** Boxplot comparing the distribution of **A**) animal fat and **B**) meat and bone meal in the feed meal received by the sow farms with and without PRRSV records in 2020 (in red the result from the logistic regression).


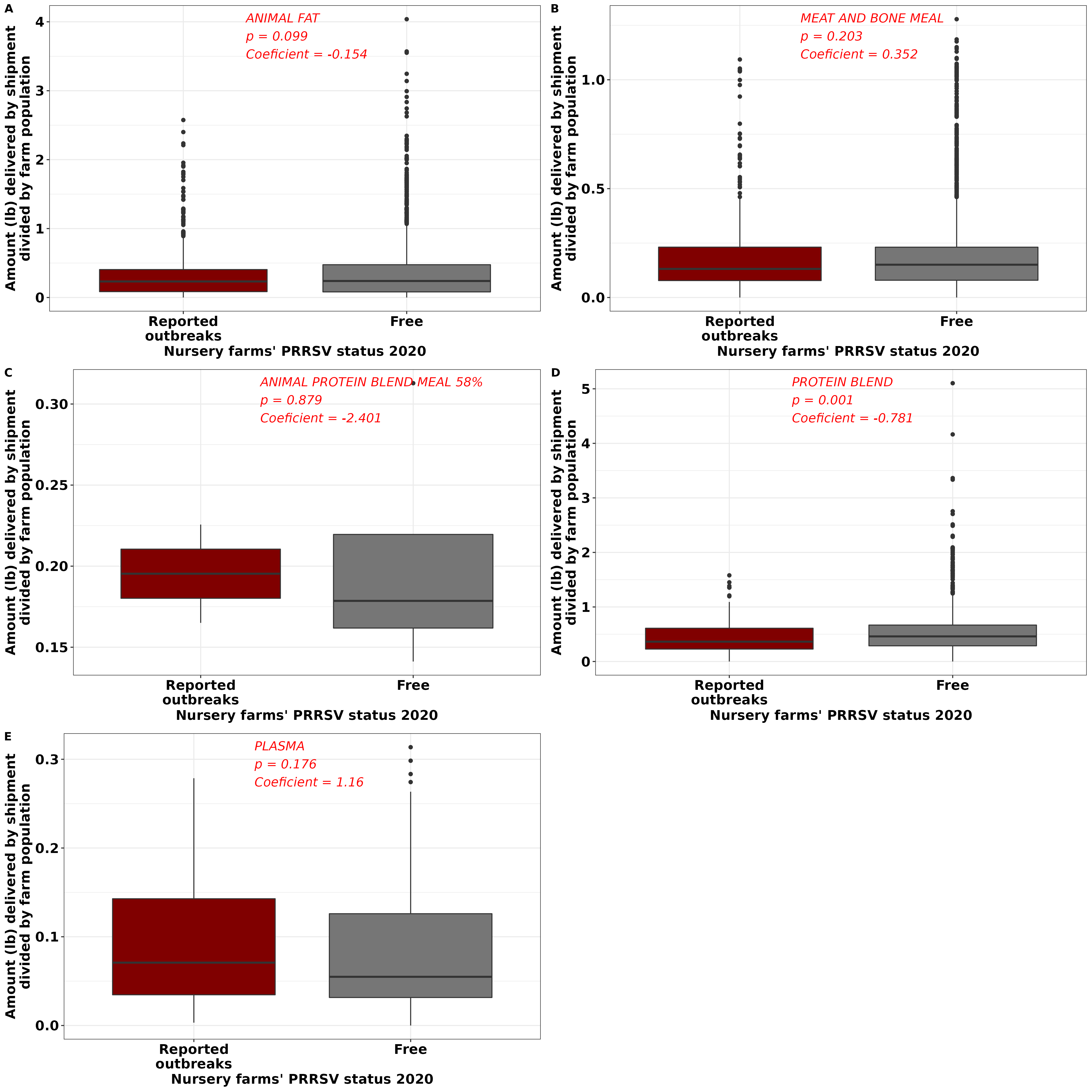


**Figure S20.** Boxplot comparing the distribution of **A**) animal fat and **B**) meat and bone meal, **C**) Protein blend meal 58%, **D**) Protein blend and **E**) plasma in the feed meal received by the nursery farms with and without PRRSV records in 2020 (in red the result from the logistic regression).


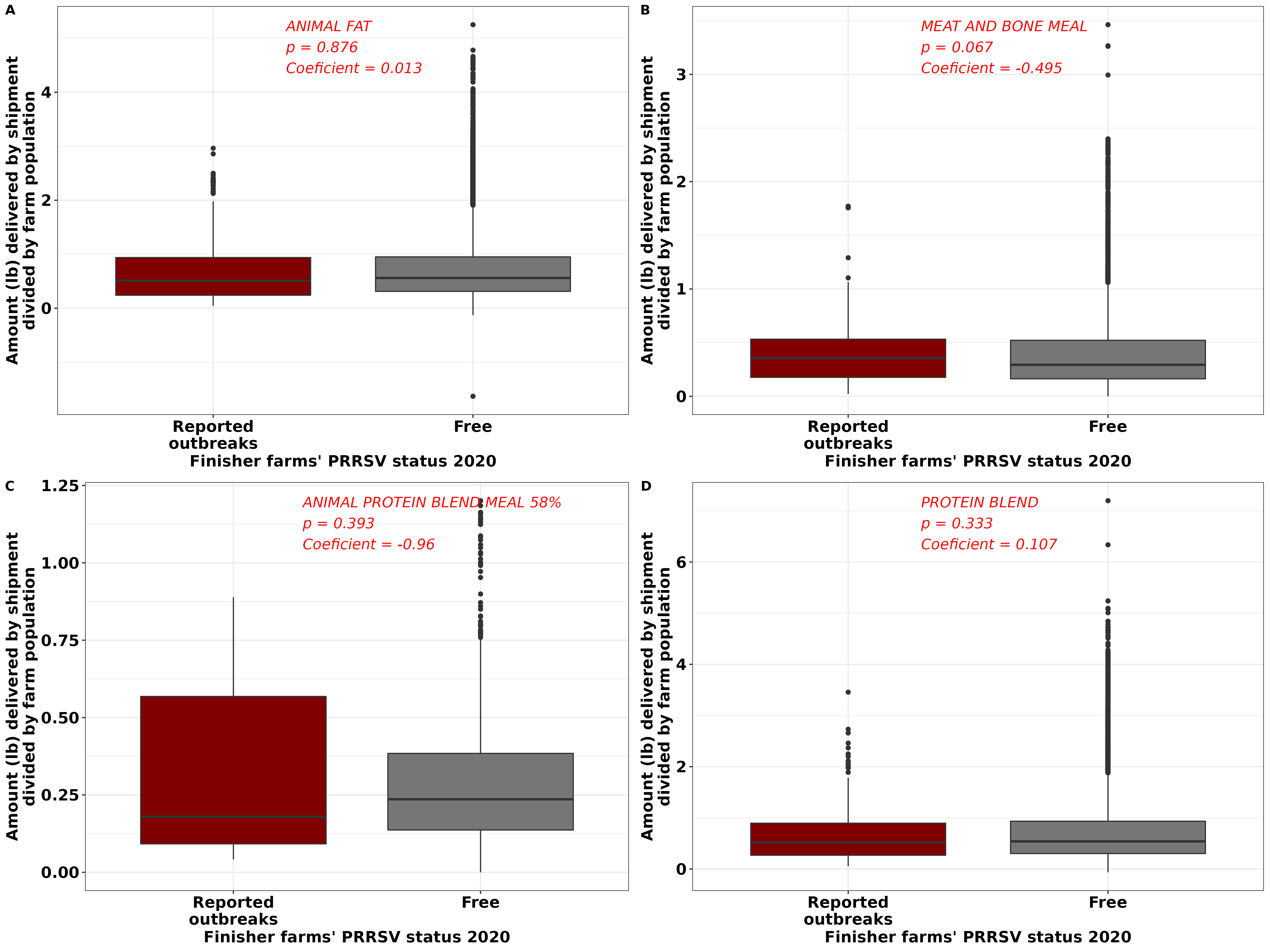


**Figure S21.** Boxplot comparing the distribution of **A**) animal fat and **B**) meat and bone meal, **C**) Protein blend meal 58% and **D**) protein blend in the feed meal received by the finisher farms with and without PRRSV records in 2020 (in red the result from the logistic regression).

***Section 4:contribution of the transmission routes.***

For company B, re-break was the main source of farm infections in sow farms with a contribution of 42.6% (95% CI 0%-93%) to PRRSV transmission, followed by local transmission with 28.1% (95% CI .8%-88%) and pig movements with 27.3% (95% CI 0%-84%), for nursery 81.6% (95% CI 31%-96%) of PRRSV transmission was related to pig movements and 18.3% (95% CI 4%-68%) to local transmission, while in finishers 51.6% (95% CI 0%-83%) was related to pig movements and 48.4% (95% CI 16%-100%) to local transmission (Figure S22). Finally, for farms of company C, local transmission was the most important transmission route for sow farms contributing with 68.9% (95% CI 8%-97%) of the farm infections, followed by re-break with 26.1% (95% CI 0%-45%) and pig movements 1.2% (95% CI 0%-0%), in nursery 53.2% was related to local transmission (95% CI 8%-100%) and 46.8% (95% CI 0%-92%) to pig movements, and for finishers 78% was related to local transmission (95% CI 31%-100%) and 22% (95% CI 0%-69%) to pig movements (notice that the contribution of transmission routes from the same company and farm type may not reach 100% because for some weeks none of the transmission routes used in the simulations was able to infect farms) (Figure S22).


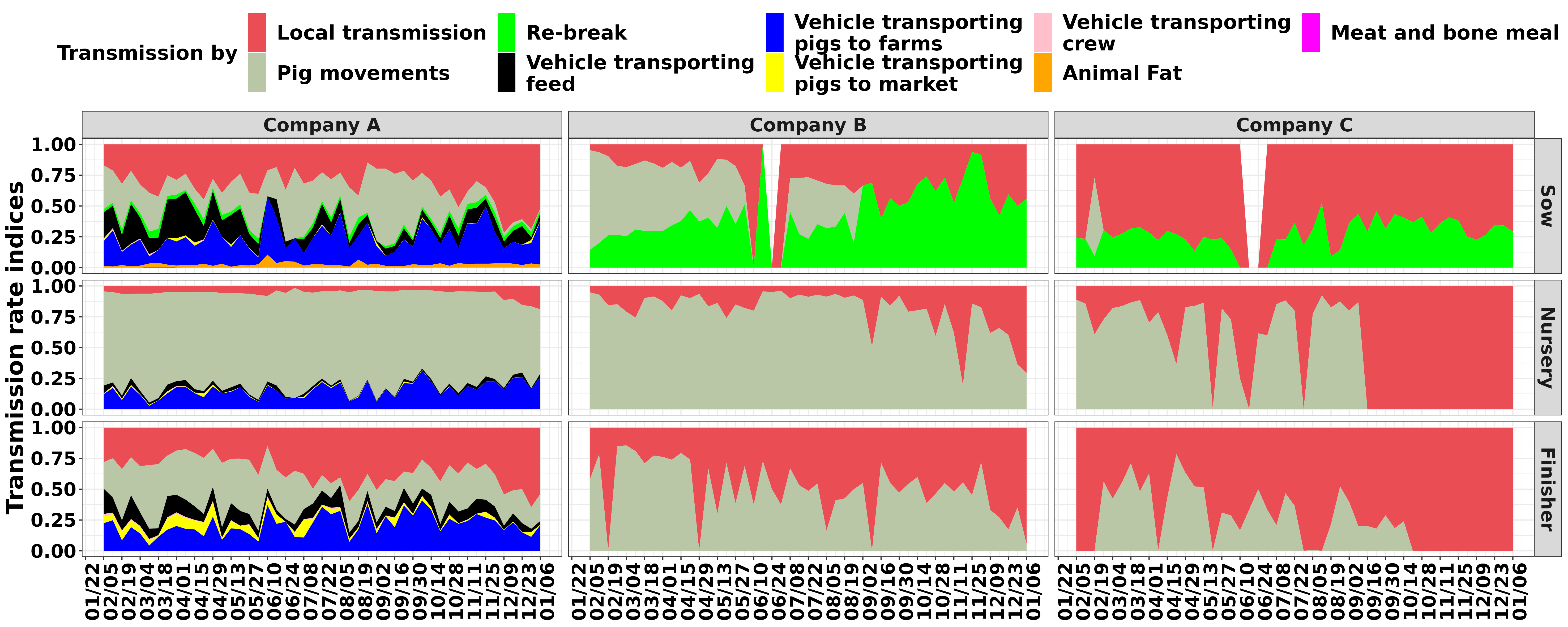


**Figure S22. Farm infection contribution for each transmission route of each company (columns) and farm types (rows).** The *y*-axis represents the proportion of transmission by each transmission route, while the x-axis shows each week in the simulation. Weekly proportions of transmission were calculated by dividing the number of simulated infected farms for each transmission route by the number of simulated infected farms for the total number of routes combined. White areas represent weeks without farm infections.

**References**

Freeman, L.C., 1978: Centrality in social networks conceptual clarification. *Soc. Netw.* **1**, 215–239, DOI: 10.1016/0378-8733(78)90021-7.

Kao, R.R., D.M. Green, J. Johnson, and I.Z. Kiss, 2007: Disease dynamics over very different time-scales: foot-and-mouth disease and scrapie on the network of livestock movements in the UK. *J. R. Soc. Interface* **4**, 907–916, DOI: 10.1098/rsif.2007.1129.

Lentz, H.H.K., A. Koher, P. Hövel, J. Gethmann, C. Sauter-Louis, T. Selhorst, and F.J. Conraths, 2016: Disease Spread through Animal Movements: A Static and Temporal Network Analysis of Pig Trade in Germany. (Thierry Boulinier, Ed.)*PLOS ONE* **11**, e0155196, DOI: 10.1371/journal.pone.0155196.

Nöremark, M., and S. Widgren, 2014: EpiContactTrace: an R-package for contact tracing during livestock disease outbreaks and for risk-based surveillance. *BMC Vet. Res.* **10**, 71, DOI: 10.1186/1746-6148-10-71.

Wasserman, S., and K. Faust, 1994: Social Network Analysis: Methods and Applications. Cambridge ; New York: Cambridge University Press.
